# Historical changes in plant water use and need in the continental United States

**DOI:** 10.1371/journal.pone.0256586

**Published:** 2021-09-02

**Authors:** Michael T. Tercek, David Thoma, John E. Gross, Kirk Sherrill, Stefanie Kagone, Gabriel Senay

**Affiliations:** 1 Walking Shadow Ecology, Gardiner, Montana, United States of America; 2 US National Park Service, Inventory and Monitoring Program, Fort Collins, Colorado, United States of America; 3 US National Park Service, Climate Change Response Program, Fort Collins, Colorado, United States of America; 4 U.S. Geological Survey (USGS), Earth Resources Observation and Science (EROS) Center, North Central Climate Adaptation Science Center, Fort Collins, CO, United States of America; 5 ASRC Federal Data Solutions, contractor to the USGS EROS Center, Sioux Falls, SD, United States of America; UNAM, MEXICO

## Abstract

A robust method for characterizing the biophysical environment of terrestrial vegetation uses the relationship between Actual Evapotranspiration (AET) and Climatic Water Deficit (CWD). These variables are usually estimated from a water balance model rather than measured directly and are often more representative of ecologically-significant changes than temperature or precipitation. We evaluate trends and spatial patterns in AET and CWD in the Continental United States (CONUS) during 1980–2019 using a gridded water balance model. The western US had linear regression slopes indicating increasing CWD and decreasing AET (drying), while the eastern US had generally opposite trends. When limits to plant performance characterized by AET and CWD are exceeded, vegetation assemblages change. Widespread increases in aridity throughout the west portends shifts in the distribution of plants limited by available moisture. A detailed look at Sequoia National Park illustrates the high degree of fine-scale spatial variability that exists across elevation and topographical gradients. Where such topographical and climatic diversity exists, appropriate use of our gridded data will require sub-setting to an appropriate area and analyzing according to categories of interest such as vegetation communities or across obvious physical gradients. Recent studies have successfully applied similar water balance models to fire risk and forest structure in both western and eastern U.S. forests, arid-land spring discharge, amphibian colonization and persistence in wetlands, whitebark pine mortality and establishment, and the distribution of arid-land grass species and landscape scale vegetation condition. Our gridded dataset is available free for public use. Our findings illustrate how a simple water balance model can identify important trends and patterns at site to regional scales. However, at finer scales, environmental heterogeneity is driving a range of responses that may not be simply characterized by a single trend.

## Introduction

Within topoedaphic constraints, climate largely controls the distribution of vegetation, which in turn determines energy, nutrient and water cycles—as well as biodiversity–in natural ecosystems [1–3]. For these reasons, climate change will continue to affect the U.S. national park system [1,2] in ways that are critical to its mission of conserving nature. Most US National parks are experiencing temperatures that are extremely warm relative to historical observations [4–6], but temperature and precipitation often have weak correlations with trends in natural resources [7,8]. Water balance variables, in contrast, are often more proximal to the mechanisms that affect plants and animals, making them more representative of ecologically-significant changes [9]. For example, Climatic Water Deficit (CWD, evaporative demand not met by available water) is more strongly related to wildfire [10] than precipitation because CWD, a measure of dryness, integrates the timing of precipitation events with fluctuating, temperature-driven evaporation. Similarly, estimated soil moisture is more strongly related to vegetation growth than precipitation because it serves as a storage reservoir between rain events [11]. Furthermore, temperature and precipitation can have counterintuitive effects on resources when considered in combination, but many of these interactive effects can be elucidated by water balance variables. Not all precipitation is available for plants when temperatures are too cold for growth, and not all precipitation becomes available to plants with warmer temperatures because of losses to evaporation and runoff. Plant water use, however, is highly correlated with Actual Evapotranspiration (AET; total evaporative water loss to the atmosphere) across a wide range of temperature and precipitation regimes [9].

A robust method for characterizing the biophysical environment of terrestrial vegetation uses the bivariate relationship between AET and CWD. These variables can be calculated from temperature and precipitation and are more easily estimated from a water balance model than measured directly [9]. Water balance models vary in form and complexity [12,13], but all track the *interaction* of primary meteorological measurements through time, as well as their mediation by topoedaphic properties [14,15]. CWD and AET have been used to effectively predict the types of vegetation that occur over scales representing ten orders of magnitude [9], and they are conceptually useful because they distinguish between different types of ‘dry’ and ‘wet’ conditions in a plant-centric way. For example, the ‘dry’ experienced by plants on sunny slopes (high CWD, high AET) differs from the ‘dry’ experienced by plants growing on soils with low water holding capacity (high CWD, low AET) [3,9]. The driver causing the former type of drought is temperature-driven evaporative demand that outstrips a potentially large soil moisture reservoir, while the driver of the latter is a small water supply, which can cause dry conditions even in moderate temperatures. Different kinds of dryness result in plant communities with different adaptive traits [9,15]. Because AET and CWD are more mechanistically related to ecological processes than temperature and precipitation, their statistical relationships with natural resources often are not only stronger but exhibit more stationarity than relationships with temperature. For example, as climate change progresses, historically strong relationships between temperature and plant species distribution can become decoupled, whereas statistical relationships between CWD and vegetation remain significant [16–18].

Here we evaluate trends and spatial patterns in AET and CWD in the Continental United States (CONUS) during 1980–2019, using a water balance model developed by Thornthwaite [19] that has been particularly influential in ecological studies [20]. The model’s relative simplicity allows it to be applied over a broad range of environmental conditions and in situations where sparse data availability prohibits the use of more complex models [21,22]. Recent studies have successfully applied variations of Thornthwaite’s water balance model to fire risk and forest structure in both western and eastern U.S. forests [8,23,24], arid-land spring discharge [25], amphibian colonization and persistence in wetlands [26–28], whitebark pine mortality and establishment [29–31], and the distribution of arid-land grass species and landscape scale vegetation condition [7,8,32,33].

We describe CONUS-wide changes in AET and CWD because national parks, the focus of our team’s research, are managed within a broader landscape consisting of neighboring public and private lands [34–36]. Park management options are often contingent on the surrounding context. After describing broad, geographical patterns of historical changes in AET and CWD, we present an illustrative example from Sequoia National Park, which highlights the variability in AET and CWD that is often observed on a smaller spatial scale. The caveats revealed by this illustrative example will help guide best-practice uses of the gridded water balance dataset described here, which is available for public use at the following URL: http://www.yellowstone.solutions/thredds/catalog.html.

## Methods

### Water balance and climate data

We estimated water balance using methods first developed by Thornthwaite [19,37] with modifications as described below. The model tracks the fate of precipitation as runoff, storage, or evapotranspiration on a daily basis. After precipitation (snow or rain) is either partially intercepted by or penetrates the vegetative canopy, it has three options: (1) remain temporarily as stored snow pack or soil moisture, (2) reenter the atmosphere via evaporation or through plants via transpiration (considered together by the model), (3) become runoff after the soil water holding capacity is satisfied. Temperature determines the duration that precipitation is stored as snow and when it melts, as well as the rate of melt and evapotranspiration. The movement of water between compartments depends on the amount of energy (heat) in the system and the amount of water available. Equations used here are well-established and have been evaluated and summarized by others [23]. Our calculations were on a daily rather than monthly timestep (compare 23) so appropriate adjustments were made, such as re-calculating solar declination as an input to Potential Evapotranspiration (PET, see below) for every day of the year rather than an average for each month. The methods for calculating each variable in the model are described briefly below.

Accumulated Snow Water Equivalent (SWE, mm) is estimated using equations from Tercek and Rodman [38] with temperature coefficients for those equations provided by Jennings et al. [39]. Actual Evapotranspiration (AET) was adjusted for differences in vegetation structure and cover, based on 30-year daily Normalized Difference Vegetation Index (NDVI) values [40]. Following similar modeling efforts [41], Potential Evapotranspiration (PET, mm) was calculated using the Oudin equation [42,43], adjusted to correct for variable heat loading [44]. Climatic Water Deficit (CWD, mm) was calculated as PET–AET. Soil Water content (mm) was calculated as the amount of water left in the top meter of soil after precipitation inputs and evaporative outputs, with soil water holding capacity values from the US Natural Resources Conservation Service [45]. Runoff was calculated as the surplus from the soil layer, i.e., when daily inputs–outputs exceeded water holding capacity.

We used temperature and precipitation from Daymet Daily Data version 3 [46–48] as model inputs. The water balance model was computed at a daily time step from 1980–2019 at 1 km resolution for the CONUS. Daily AET and CWD were summed for analyses presented here that used annual time steps. The analytical code was written in Python version 3.6 [49] using the NumPy and SciPy libraries [50] and computed as parallel processes on the Amazon cloud. The source code is available from the authors.

Here, we focus on estimates of AET and CWD. Complete daily results for all variables in the model at 1 km resolution (3 TB) can be accessed from a THREDDS server located at http://www.yellowstone.solutions/thredds/catalog.html.

### Analyses

Geographic patterns of 1981–2019 average annual CWD and AET were depicted on maps and scatterplots. Every pixel in CONUS was classified by its geographic location within US Environmental Protection Agency Level I ecoregions (https://www.epa.gov/eco-research/ecoregions; [51]), which are defined by their dominant vegetation. AET vs CWD scatter plots of the pixels within these ecoregions were compared to the geographic distribution of pixels classified according to their AET and CWD values, without regard to ecoregion membership.

We calculated trends in total annual AET and CWD for every 1 km pixel in CONUS using linear least squares regression. Regression significance was not considered because we were interested only in broad spatial patterns of change rather than making ecological inferences. Since the magnitude of change indicated by the regression slope (mm/year) may be more ecologically significant in areas with small starting values for AET and CWD, we calculated a normalized index of change per decade for each pixel as

NormalizedIndex=(regressionslopeinmmperyear/1981–2019mean)*10. (1)

This can be interpreted as the percent change relative to the mean per decade.

The annual totals of AET and CWD for each pixel were averaged for 1980–1999 and 2000–2019. The two average values for each time period were then used as endpoints of vectors in two dimensional space defined with CWD on the horizontal axis and AET on the vertical axis. The intensity of the combined change in AET and CWD (length of the vector) was calculated as the Euclidean distance between the two points defined by the two time period averages. These “change vectors” (intensity and direction) were calculated for every pixel in CONUS. Change vectors, calculated with the same method, were also plotted separately for points extracted from the centroids of all the national parks in CONUS.

## Results

### CONUS patterns in AET and CWD

**Fig 1:**
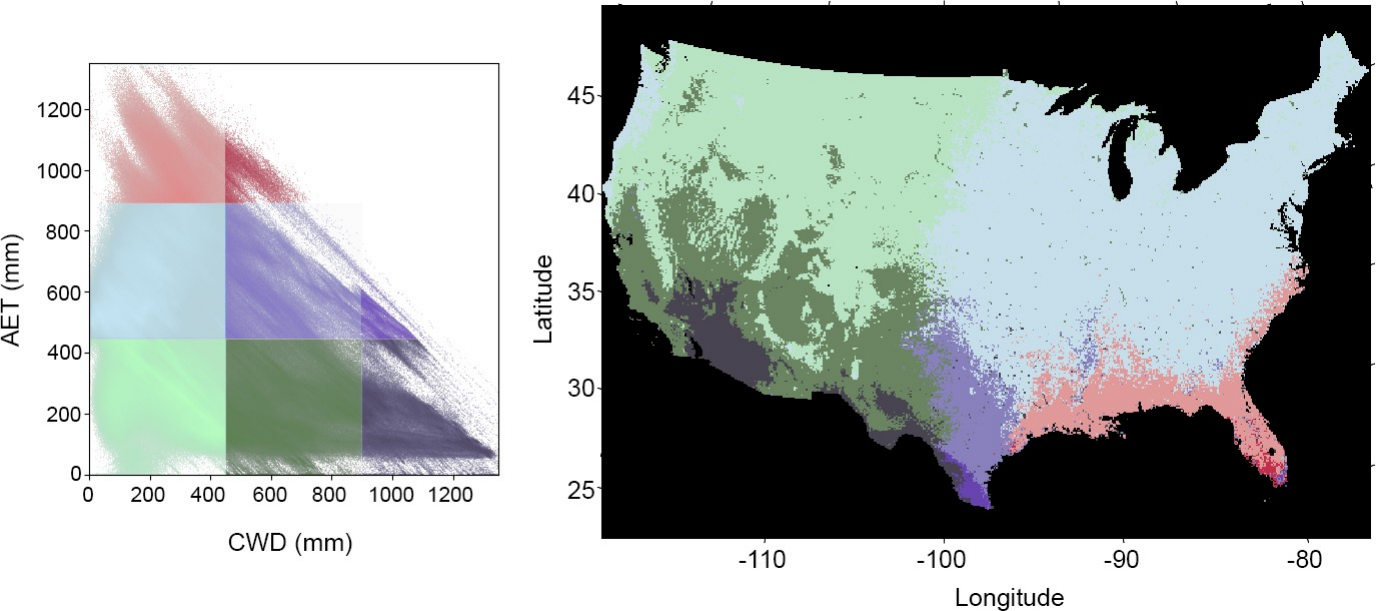
Geographic patterns in Actual Evapotranspiration (AET) and Climatic Water Deficit (CWD). LEFT: Scatterplot of 1981–2019 average annual CWD vs. 1981–2019 average annual AET for all locations in CONUS. The shading/intensity of colors indicates point density. Colors indicate equal-area zones in the AET-CWD bivariate plot; these are depicted on the map in the right panel. RIGHT: Map of CONUS showing the geographic locations of colored areas in the scatter plot. The AET vs CWD relationship has a well-established link to the distribution of dominant vegetation types (e.g., Stephenson [9]).

Across the entire CONUS, 1980–2019 average annual CWD generally increased from north to south, i.e. increased with decreasing latitude (Fig 1). Cutting across this north–south gradient was a pattern of increasing average annual AET from west to east. Areas east of the 100^th^ meridian generally had average annual AET > 400 mm, while areas west of this divide had annual AET < 400 mm (Fig 1). As expected, the most desert-like conditions of low AET and high CWD occurred in southern California, Texas, and Arizona (Fig 1, dark gray areas), and the most mesic conditions of low CWD and high AET occurred in Florida (Fig 1, reds).

**Fig 2:**
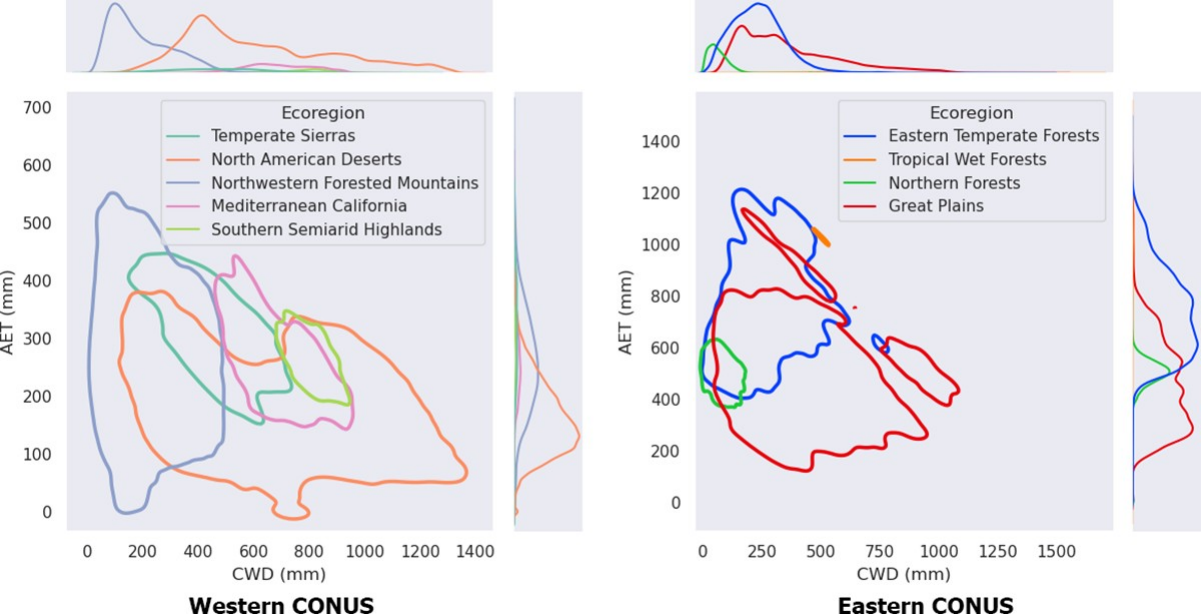
Comparison of EPA Ecoregions vs. modeled Actual Evapotranspiration (AET) and Climatic Water Deficit (CWD). LEFT: Model pixels west of the 100^th^ meridian categorized by their geographic location within EPA Level I ecoregions (https://www.epa.gov/eco-research/ecoregions; [51]) and plotted in bivariate space: 1981–2019 average annual CWD vs. 1981–2019 average annual AET. For clarity of presentation, kernel density estimators (KDEs) were used to calculate outlines for the cluster of points in each ecoregion. Right: Model pixels east of the 100^th^ meridian, categorized according to EPA ecoregions, and plotted in the same way. Marginal plots (top row, LEFT and RIGHT) show the probability density functions of AET and CWD for each ecoregion. Points from the Marine West Coast ecoregion were too sparse to create a KDE polygon, but individual points from this region appear in Fig 8.

Pixels classified into EPA ecoregions had a large degree of overlap in AET: CWD bivariate space (Fig 2). Pixels from within a single ecoregion clustered together, with ecoregions such as Tropical Wet Forests of Florida appearing where they would be expected, with high AET and low–mid CWD (compare orange areas in Fig 2 RIGHT, to dark red in Fig 1). Similarly, North American Deserts had the highest CWD and lowest AET west of the 100th meridian (Fig 2, LEFT). In neither the east nor the west, however, were any of the ecoregions distinguished by occupying a unique, non-overlapping position in the AET: CWD plots (Fig 2).

### Trends in CWD

**Fig 3:**
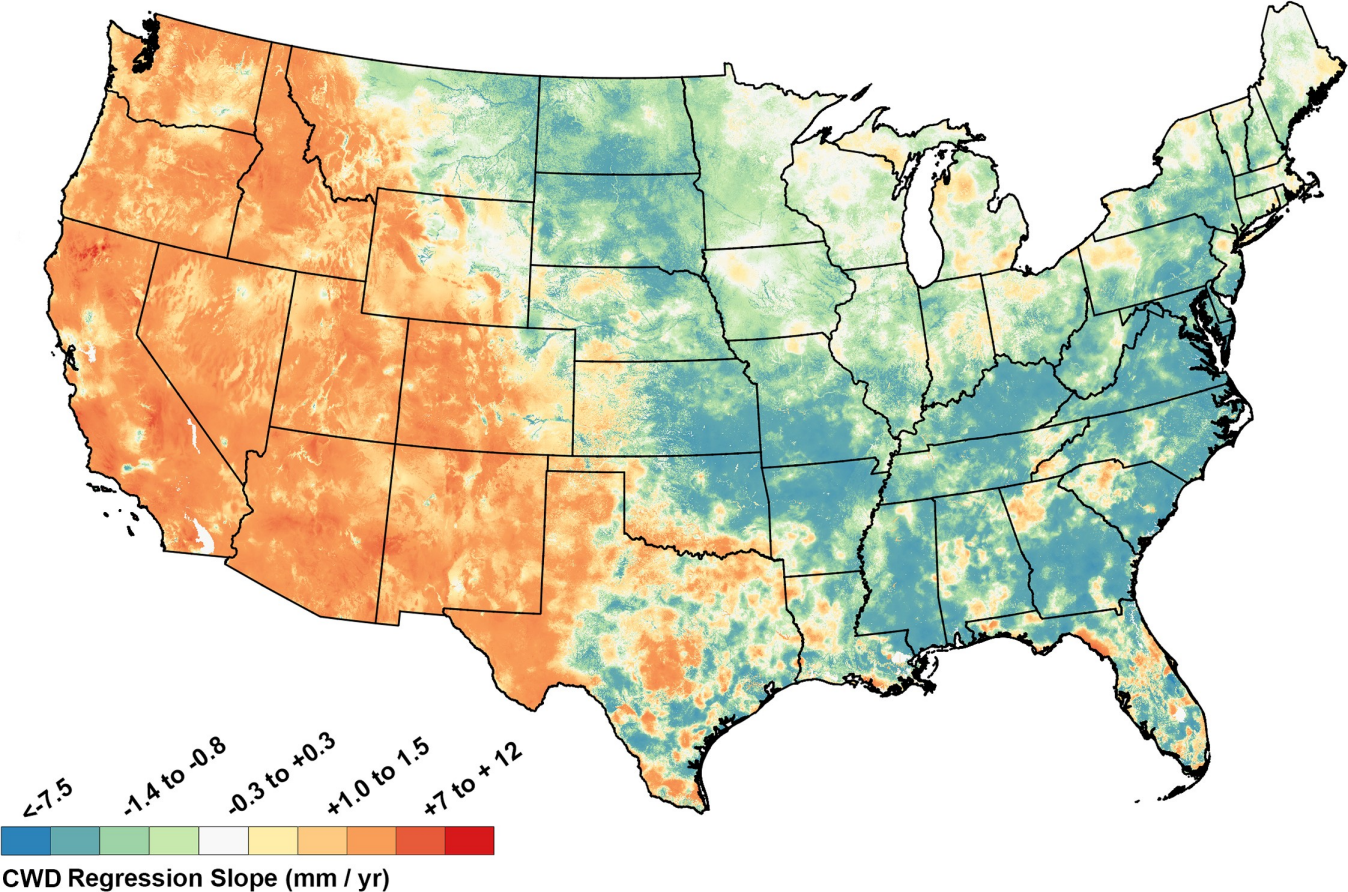
Change in annual total Climatic Water Deficit (CWD; mm/year) estimated for the period 1980–2019. Positive slopes indicate increasing CWD and consequently drier conditions.

**Fig 4:**
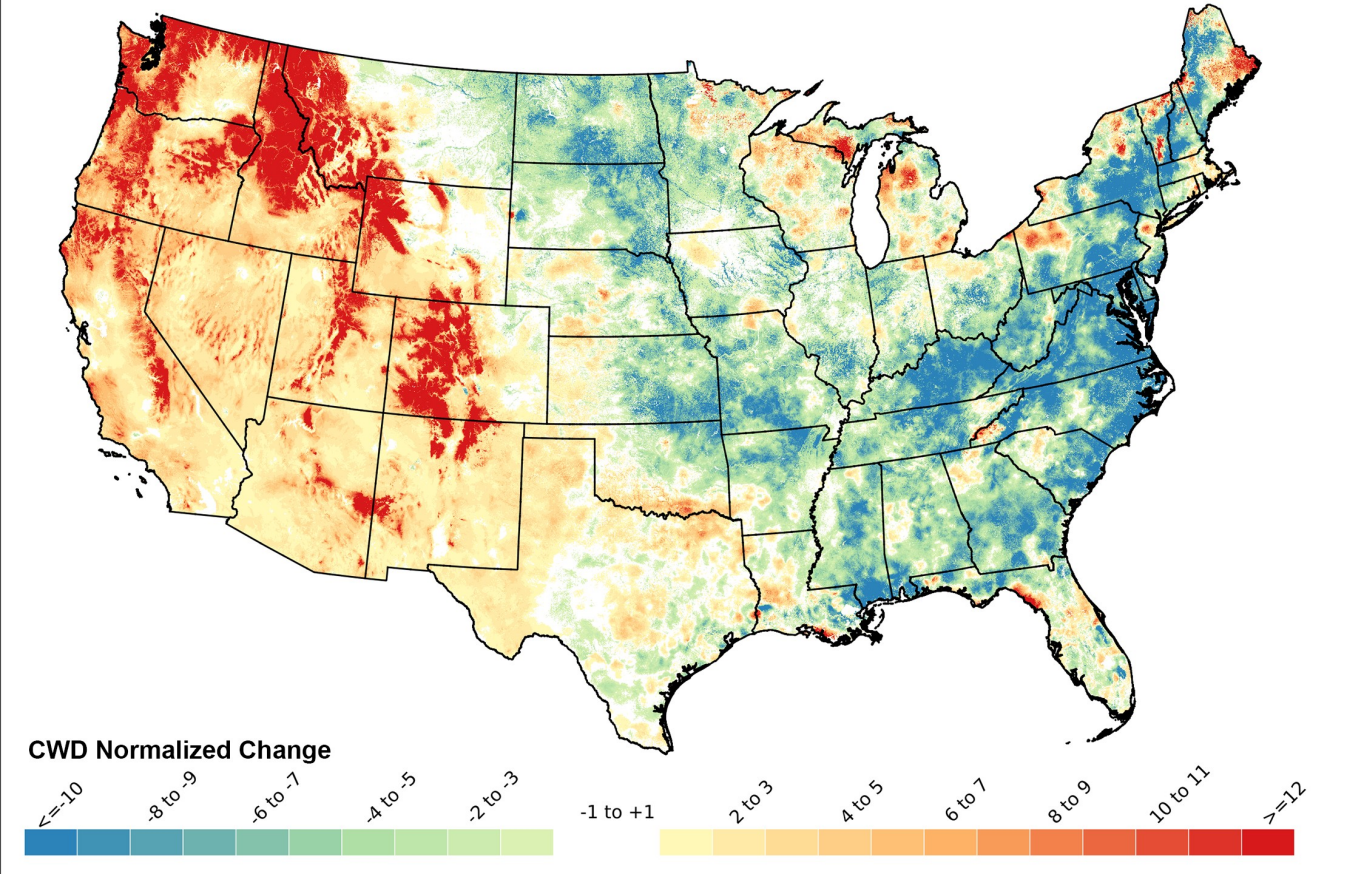
Normalized change (percent of historical mean per decade) in total annual Climatic Water Deficit (CWD), calculated as (regression slope/1980–2019 mean CWD) * 10. Regression slopes appear in Fig 3.

In general, the western US from the west coast to the Rocky Mountains, and extending south to the Texas panhandle, had linear regression slopes indicating increasing CWD (drying), while the eastern US generally had trends toward lower CWD (wetter conditions), and mixed trends were found in the Great Lakes and central regions (Fig 3). When these regression slopes were normalized relative to the mean CWD value for each pixel and expressed as percent change per decade relative to the 1981–2019 mean, it became apparent that larger relative increases in CWD (drying) had occurred particularly in the central and northern Rocky Mountains, the Pacific Northwest, and the Sierra Nevada Mountains (red areas, Fig 4). Normalized rates of change also identified that rapid decreases in CWD (reduced evaporative demand, wetter conditions) occurred in mid-latitude portions of the eastern seaboard and portions of New England (blue areas, Fig 4). The differences between Figs 3 and 4 are due to the fact that the normalized changes in Fig 4 emphasize areas in which the slopes of the regressions are larger relative to the average values of the variable at that location, whereas changes in Fig 3 are absolute rates of change.

### Trends in AET

**Fig 5:**
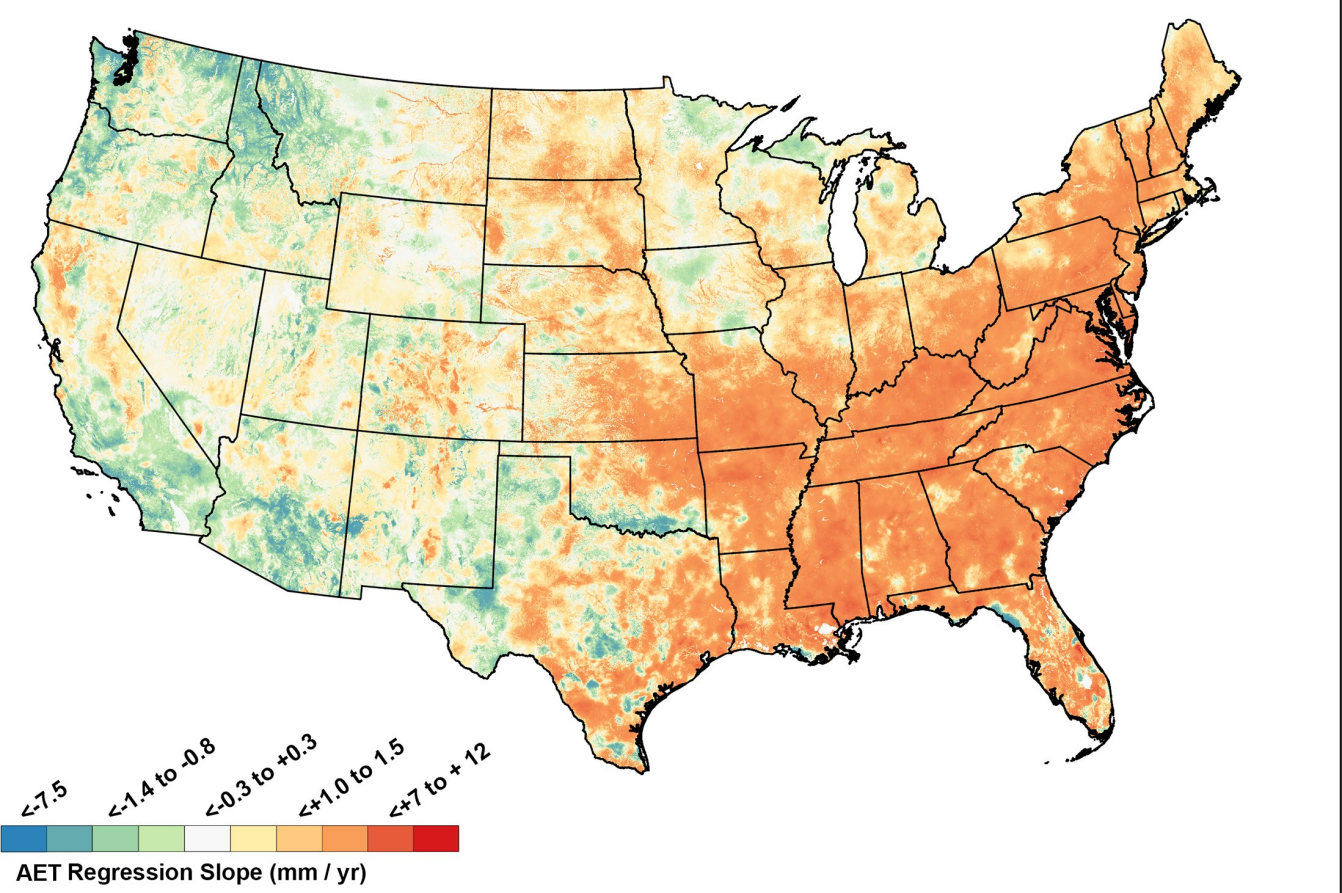
Change in annual total Actual Evapotranspiration (AET) expressed as slopes (mm/year) for the period 1980–2019. Positive regression slopes indicates increasing AET.

**Fig 6:**
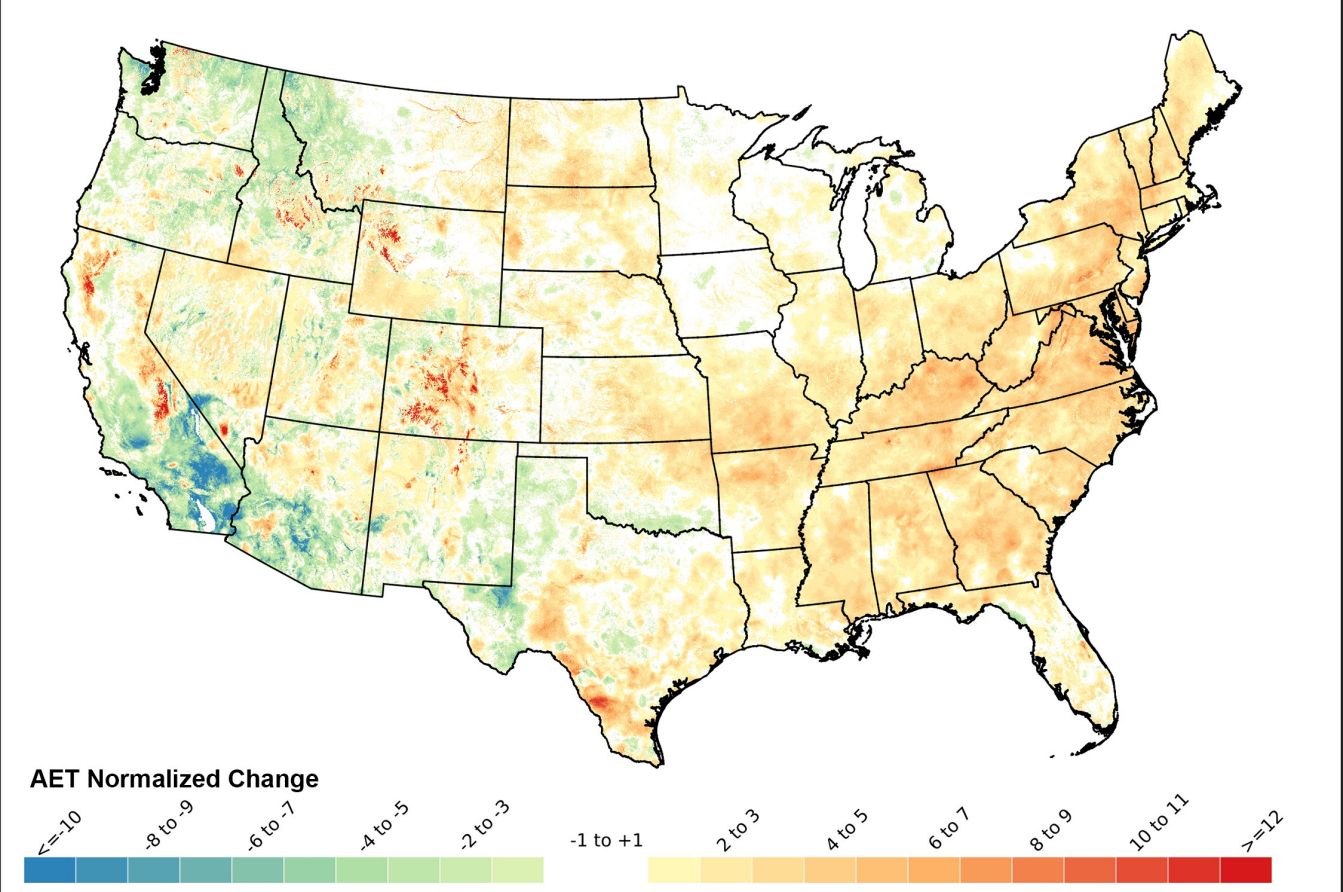
Normalized change (percent of historical mean per decade) in total annual Actual Evapotranspiration (AET), calculated as (regression slope/1980–2019 mean AET) * 10. Regression slopes appear in Fig 5.

The map of AET regression slopes (Fig 5) is to some extent a mirror image of the CWD regression map (Fig 3). Eastern parts of CONUS often had increasing AET (positive slopes; Fig 5), corresponding in many cases to locations with decreasing CWD. Western CONUS often had decreasing AET (negative regression slopes, Fig 5), corresponding to locations with increasing CWD. Middle longitudes had mixed trends for AET (Fig 5). When AET regression slopes were normalized to percent change per decade, some higher elevation areas in the west (e.g., parts of Wyoming, Colorado and the Sierra Nevada Range) had large relative increases in AET (Fig 6). In some cases, e.g. northwestern Wyoming, these areas of large normalized increases in AET were in high elevation locations with small changes in CWD (slopes near zero). Some of the greatest relative decreases in AET occurred in desert areas of southern California (Fig 6). These-water limited locations were becoming even more arid.

### Combined change in AET and CWD

**Fig 7:**
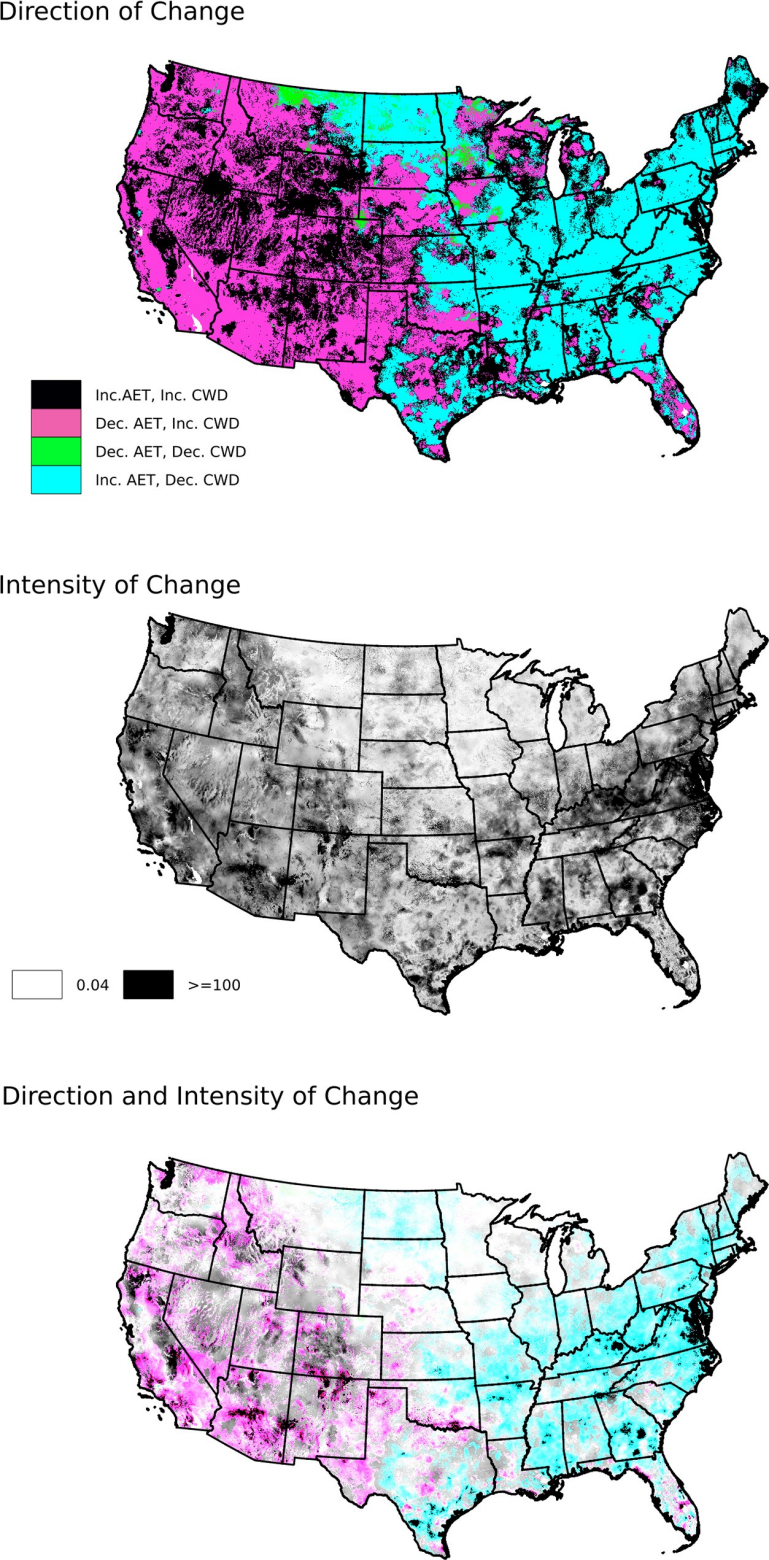
Combined (bivariate) change in total annual Actual Evapotranspiration (AET) and total annual Climatic Water Deficit (CWD) calculated from 1980–1999 and 2000–2019 means. TOP: Direction of change (increasing or decreasing for each parameter). MIDDLE: Intensity of change (mm), calculated as the Euclidean Distance (length of vector in AET:CWD space) between the two period means. BOTTOM: Illustration of combined direction and intensity of change, Colors in the bottom panel are the same as shown in the legend for the top panel, with the intensity (brightness) of each pixel determined by the shading in the middle panel.

In general, the eastern CONUS experienced the combined effects of increasing AET and decreasing CWD (became wetter and had more evapotranspiration), while western regions experienced overall drying, with increases in CWD and either increases or decreases in AET (Fig 7, Top). The intensity of this combined change was greatest in central portions of the east and in the southwest, and least in the central northern plains (Fig 7, middle). Combining the intensity and direction of change in AET and CWD (Fig 7, bottom) highlights the relatively small changes that occurred in the northern plains.

### AET: CWD change for national parks in CONUS

**Fig 8:**
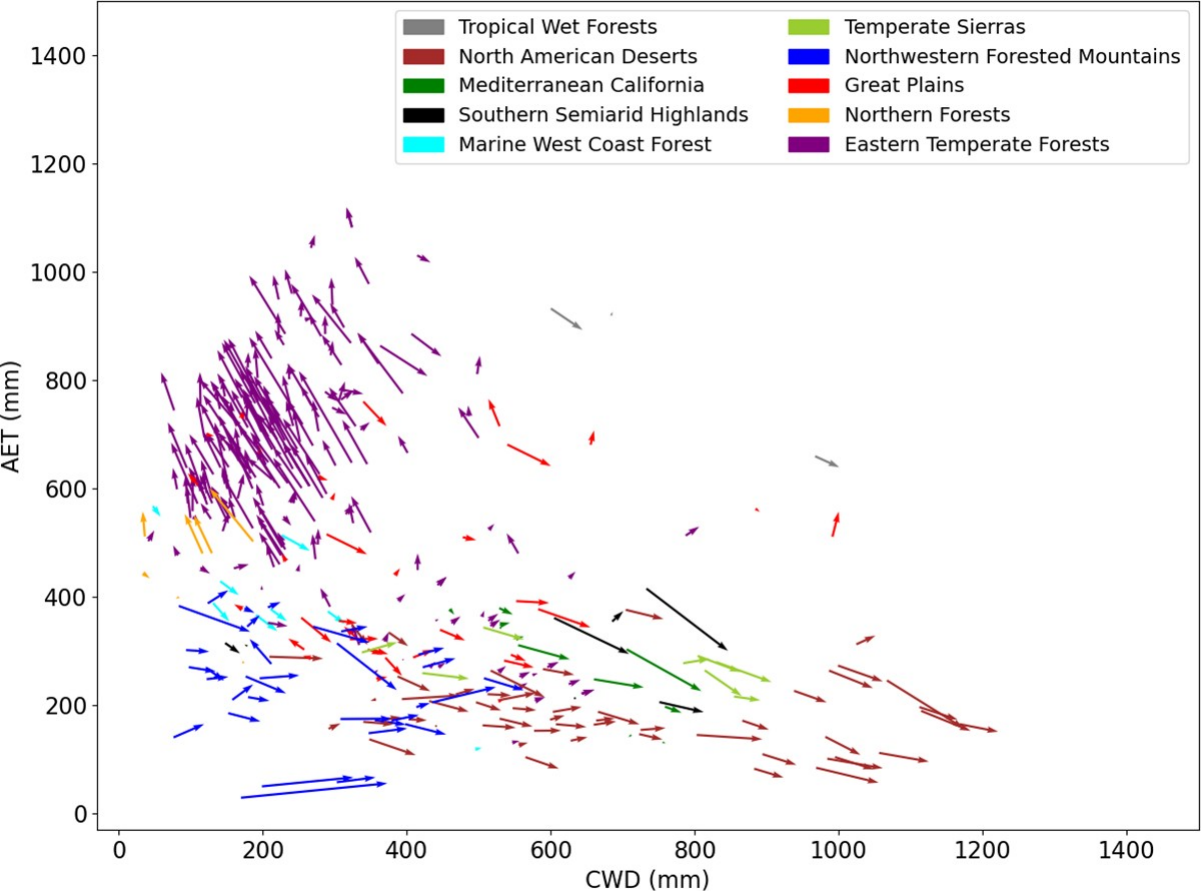
Combined change in Actual Evapotranspiration and Climatic Water Deficit for all CONUS National Park Service Units calculated from 1980–1999 and 2000–2019 means. One centroid point for each park was selected, creating one vector for each park. Parks were categorized (different colors) by EPA Level 1 Ecoregion.

Vectors showing the movement of CONUS national parks in AET: CWD space (Fig 8) provide localized snapshots of the patterns shown in Fig 7. In general the wettest parks got wetter and the driest parks got drier. For example, parks in the Eastern Temperate Forest ecoregion, which plotted farthest to the top-left of the AET: CWD plot, generally moved even further in that direction, increasing their average annual AET and decreasing their average annual CWD (Fig 8). Conversely, parks in the North American Deserts, which plotted bottom–right, moved further in that direction, increasing CWD and decreasing AET (Fig 8).

## Discussion

### Geographic patterns of AET: CWD and change during the study period

The primary driver for AET:CWD trends in the western U.S. (Figs 3 and 5) was increasing temperature, while precipitation played a more dominant role in the east. CWD generally increased and AET decreased in the western US because western temperature increases during the study period were steeper than in the east and western precipitation trends were usually flat or declining [52–54, S1–S4 Figs]. In contrast, the eastern United States often had both higher average annual precipitation than the west during the study period and often increasing precipitation trends, which allowed for increased AET and declining CWD [53–56] (Figs 3, 5 and S1–S4).

The broad geographic patterns of AET:CWD (Fig 1) are congruent with the temperature and precipitation patterns just described, but the distribution of EPA ecoregions in our bivariate plots did not provide the separation we expected, given the many studies that have used these variables to explain vegetation distribution [9, others cited in introduction]. Higher average precipitation in the east [53–56] contributed to that region’s greater AET, and warmer temperatures in the south contributed to the north—south pattern of increasing CWD (Fig 1). The failure of the EPA ecoregions to plot discretely suggest a high degree of heterogeneity at the Level I ecoregion scale. That is, they are large enough to encompass high vertical relief or cross-latitude gradients that span a range of AET and CWD. We illustrate these points with a closer look at Sequoia National Park, in the Sierra Nevada mountains of California.

### Fine scale changes in Sequoia National Park

**Fig 9:**
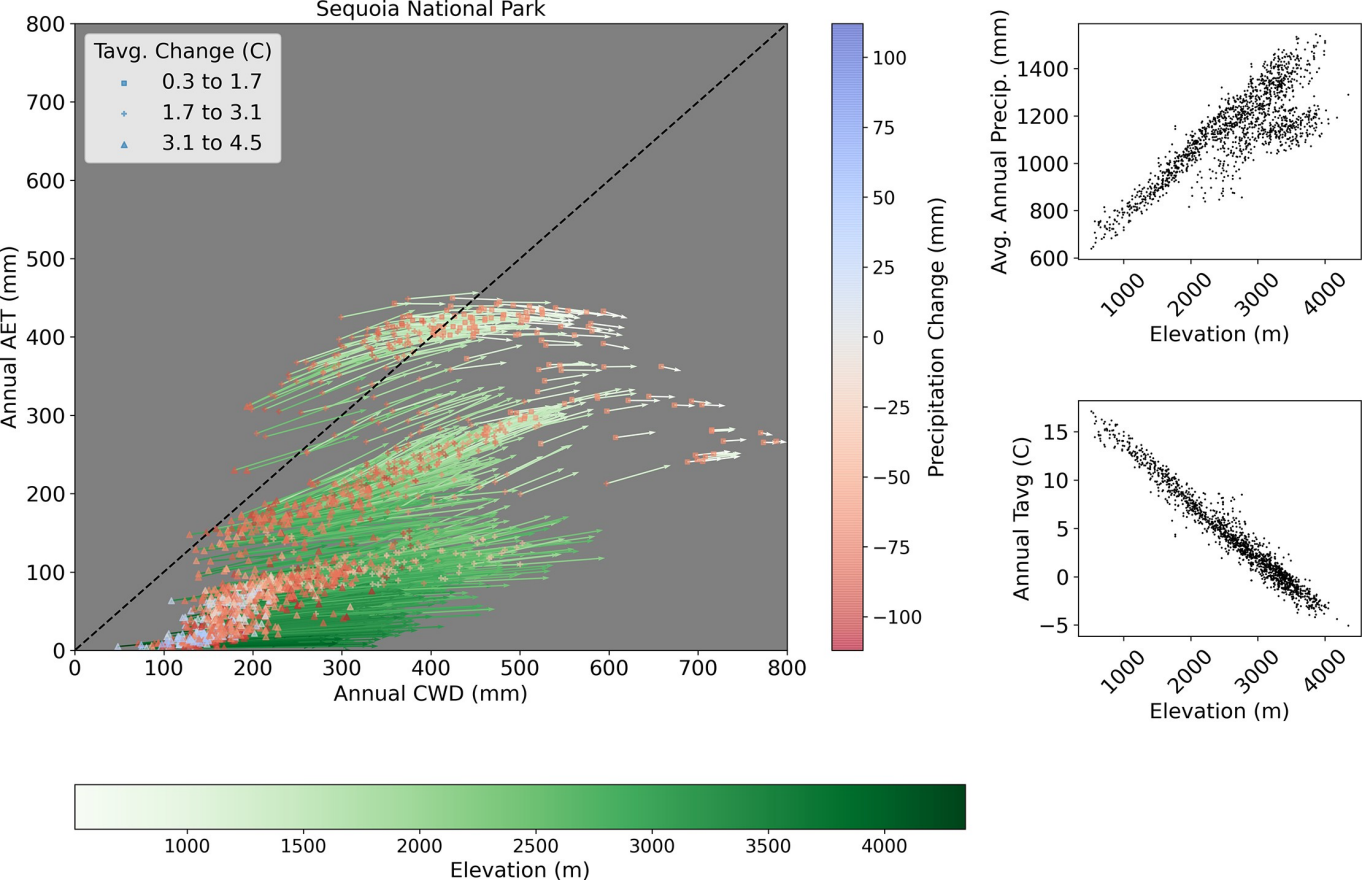
Change in bivariate climate space for Sequoia National Park. Arrows = combined change in Actual Evapotranspiration (AET) and Climatic Water Deficit (CWD) from 1980–1999 to 2000–2019 (means). Black dashed line = 1:1 demonstrating equal change in AET and CWD for comparing vector directions. Vector colors (green shades) indicate elevation. Points (squares, pluses, triangles) are located at the starting point of every vector and indicate amount of temperature change between the two time periods, with colors of the points indicating the amount of change in average total annual precipitation between the two time periods. Insets show the strong correlations between elevation and average temperature/precipitation during the entire study period.

Pixels within and around Sequoia National Park straddle the boundary between just two Level I EPA ecoregions: Northwestern Forested Mountains and Mediterranean California, but average annual conditions in that area range from 50 mm– 800 mm CWD and 10–450 mm AET (compare Figs 2 and 9), while vegetation types range from chaparral, to montane forests, to giant Sequoia Groves, to high alpine communities, as well as others [57]. An examination of change vectors (calculated as in Fig 8) within this region reveals that bivariate changes varied in large part along an elevation gradient spanning approximately 500 m– 4300 m (Fig 9, green shading of vectors). The greatest bivariate changes occurred at higher elevations (Fig 9, darkest green vectors), where increasing trends in temperature were greatest (Fig 9, triangles) and average annual precipitation was increasing during the study period (Fig 9, blue symbols and inset). These higher elevation changes (> 2000 m) were comprised primarily of increases in CWD and little change in AET (Fig 9). At high elevations, increase in AET is due to rising temperatures that melt snow earlier and maintain evapotranspiration longer when water is not limiting. Similarly, at high elevations CWD increases because a longer period of drying may draw down soil moisture in some years when soil water is not replenished after snowmelt. Middle elevations (Fig 9, 1000–2000 m) generally experienced moderate increases in both AET and CWD, while lower-elevation locations, where average annual precipitation was lowest and the smallest temperature increase occurred, generally had the smallest bivariate changes, and they were comprised of CWD increases with little change in AET (Fig 9).

We make two observations from this example, which shows great diversity in response to climate change within a relatively small (~1500 square kilometer) area. First, plots such as shown in Fig 2 likely would show better separation among vegetation communities if created for smaller geographic areas, using pixels classified according to vegetation maps more specific to the region, e.g. in this case including giant sequoias, alpine, etc. Second, appropriate use of our data to inform management decisions in topographically diverse area will require examining specific locations in each park or region of interest, rather than using single points to represent a region as in Fig 8. Where such topographical and climatic diversity exists, appropriate use of our gridded data will usually require sub-setting the data to an appropriate area, and analyzing it according to categories of interest such as vegetation communities or across obvious physical gradients. The existence of our pre-calculated, gridded dataset makes it possible to rapidly conduct analyses like those shown for Sequoia National Park in Fig 9. Prior to the development of this dataset, regional analyses like those presented here were time consuming and laborious [8].

### Ecological implications

Terrestrial plants grow only when they transpire, thus AET is highly correlated with plant growth, and CWD, defined as unmet water need, is synonymous with drought stress [9]. Together, these variables characterize key elements of the hydrological environment that plants experience. Vegetation growth is positively correlated with AET and negatively correlated with CWD at plot to landscape scales [7,8,33,58–60]. When limits to plant performance characterized by AET and CWD are exceeded, vegetation assemblages change [61]. Plant traits such as rooting depth and stomatal resistance interact with climate to determine competitive relationships between species and responses to climate changes [60,61]. When drought, measured by CWD, exceeds the stress tolerance of a particular species, then the decline of a species may allow the colonization or increase of other species better suited to drier conditions (sometimes non-natives) [61]. Widespread increase in aridity throughout the west, expressed by increasing CWD, portends shifts in the distribution of plants limited by available moisture. Alternatively, increases (or decreases) in AET can confer advantages (or disadvantages) to species that rely on abundant plant-available moisture and high growth rates to compete [24,58,60].

Vegetation responses to changes in AET:CWD can be abrupt, as illustrated by rapid range extensions of non-native and invasive cheat grass (*Bromus tectorum*), or gradually, as exhibited by the shifts in abundance in cool and warm season (C3 and C4) grass species [7]. Gradual vegetation shifts due to changes in AET are already taking place in eastern forests [24], while abrupt disturbance-driven events linked with increasing CWD are altering treeline forests of the western U.S. [29,62]. In the southwest US, increases in aridity indicated by increase in CWD, suggest more drought adapted species may have competitive advantage in the future. For example, juniper trees persist longer than pinyon trees under drought stress because pinyon trees stop transpiring, which limits carbon uptake [63]. Recent extreme drought in California between 2011 and 2016 killed millions of trees [64]. In many ecosystems, wildfire frequency and severity is controlled primarily by drought rather than fuel loads, particularly mid-elevation forests in the Pacific northwest [65], and acute episodes of high CWD are good indicators of conditions dry enough to burn [8]. These are some of the changes that have been documented, but there are likely other unnoticed changes taking place more slowly that we do not yet fully understand. For example, the cascading effects set off by drought stress, as indicated by CWD, can result in hydraulic failure, carbon starvation or susceptibility to disease or pests, such as mountain pine beetle [29,66,67].

### Model limitations

One source of error in our results derives from the inaccuracies inherent in the Daymet input data to our water balance model. Like all gridded climate datasets, Daymet uses lapse rates and terrain factors to interpolate weather station measurements to remote locations [68]. As a result, some areas that contain fewer weather stations, such as higher elevations, might have less accurate estimates of temperature and precipitation, which were used to drive the model [68–71]. This error was ameliorated in our analysis by our focus on trends in the water balance variables rather than their absolute magnitudes, though temperature trends calculated for higher elevations can sometimes be inflated due to problems with methods used at high-elevation weather stations [69]. Users should be aware of these issues in their research contexts [71]. Additionally, errors in the other input layers can in some cases produce unrealistic results. For example, calculations for pixels that cover lakes, straddle coastlines, or include other topographic features that have very low (or no) soil water holding capacity may provide unrealistic estimates of AET. Future research that uses the water balance results discussed here should examine multiple pixels in a region (see below for our example in Sequoia National Park), and mask points that are not representative of terrestrial conditions where plants grow, such as lakes or glaciers.

The equations used in our model have been validated by others (citations in methods above), but choices in particular aspects of the implementation can affect results. For example, the Oudin calculation of PET is less grounded in physical equations compared to the Penman-Monteith method [72], but in the absence of input parameters that are not available in gridded climate data (e.g., wind speed), methods like Penman-Monteith use approximations that reduce performance, reaching a level that is similar to or worse than more empirical methods such as Oudin [42,43,71,72]. For a demonstration of how Penman-Montieth methods converge on other methods when input data are missing see Tercek et al. [71]. Indeed, Hostetler and Alder [41] used the Oudin method to reproduce runoff at 1427 CONUS stream gages with a median Nash-Sutcliffe efficiency = 0.57. Our methods differ from Hostetler and Alder [41] only in our refinement of the equations used to calculate snow dynamics by incorporating pre-calculated temperature coefficients that account mostly for regional differences in relative humidity [38,39], and in our adjustment of AET with daily NDVI values [40] to account for broad-scale difference in vegetation structure. These two model enhancements substantially improved our estimates of annual peak SWE, number of days per year with snow cover, and the dates of seasonal start and loss of snow compared to estimates from SNODAS satellite imagery (S6 Fig). Similarly, our AET estimates have errors centered on zero difference from flux tower observations and generally decreasing numbers of grid cells with larger differences (S5 Fig).

## Conclusions

Bivariate changes in AET and CWD are well established indicators of ecological change because of their strong relationship to vegetation composition and dynamics [9,20,73]. Many US parks are experiencing temperatures outside their historic range of variation [4] and we found these temperature increases are often driving directional changes in plant-available water and water use. Water budgets, including changes in AET:CWD, are more ecologically relevant indicators of the consequences of climate variation than changes in temperature and precipitation [8,9], and they provide a more accurate estimate of climate as a driver of natural resource response [59]. In some cases, rates of bivariate changes, such as AET x CWD, are expected to be more rapid than changes in the individual variables, which makes them robust and sensitive indicators of impactful consequences of climate change [74,75]. Despite considerable uncertainty in projections of even the direction of precipitation change, there is much greater concurrence in projected trends in water balance trends for much of the water-limited drylands of the US and Canada [75].

Our findings illustrate how a simple water balance model, with enhancements to account for regional difference in vegetation cover and snow dynamics, can identify important trends in water balance variables at site to regional scales. At a coarse scale, ecosystems across the western United States are drying and in the east are becoming wetter. However, at the park scale, environmental heterogeneity is driving a range of responses that may not be simply characterized by a single trend. Our team and others have established key relationships between water balance and high-priority resource issues that include vegetation dynamics, freshwater flows, wildfire ignitions, and dynamics of water-sensitive species [7,8,25,27,33]. When water balance trends are interpreted in the context of these relationships, they can serve as important indicators of ecological change that can inform management decisions.

## Supporting information

S1 Fig Change in average annual temperature (C) 2000–2019 vs 1980–1999. Data source = Daymet.
(TIF)

S2 Fig Change in average annual precipitation (mm) 2000–2019 vs 1980–1999. Data source = Daymet.
(TIF)

S3 Fig Average annual precipitation (mm) 1980–2019. Data source = Daymet.
(TIF)

S4 Fig Average annual temperature (C) 1980–2019. Data source = Daymet.
(TIF)

S5 Fig Comparison of Snow Water Equivalent (SWE) estimates in the gridded water balance model vs SNODAS (https://nsidc.org/data/g02158) quantified as the first counted day in the water year (October 1 –September 30) with snow cover, last counted day in water year with snow +1, peak SWE (greatest water year value), and days/year with SWE > 0. Estimates for all years are averaged for 2005–2019 before comparison to the corresponding pixel. Differences are calculated as model average–SNODAS average.
(PNG)

S6 Fig Comparison of Actual Evapotranspiration (AET) estimates from the CONUS gridded water balance model and AET from fluxnet stations (https://fluxnet.org). Pixels corresponding to the location of towers were extracted from the gridded dataset and classified by vegetation communities specified by fluxnet.org. Monthly differences between total AET values in the model vs at the towers were calculated over 1980–2019, or for the period of record for the flux towers, whichever was shorter. X- axis = difference in mm model AET–tower AET. Y–axis = number of months with the specified difference.
(PNG)

S7 Fig Locations of National Parks in the Continental United States (Black dots). Sequoia National Park is marked with a purple diamond.
(TIF)
