# Supplementary figures and images for: Historical changes in plant water use and need in the continental United States
Source: PLoS One. 2021 Sep 2;16(9):e0256586. doi: 10.1371/journal.pone.0256586 (PMC8412362; doi:10.1371/journal.pone.0256586)

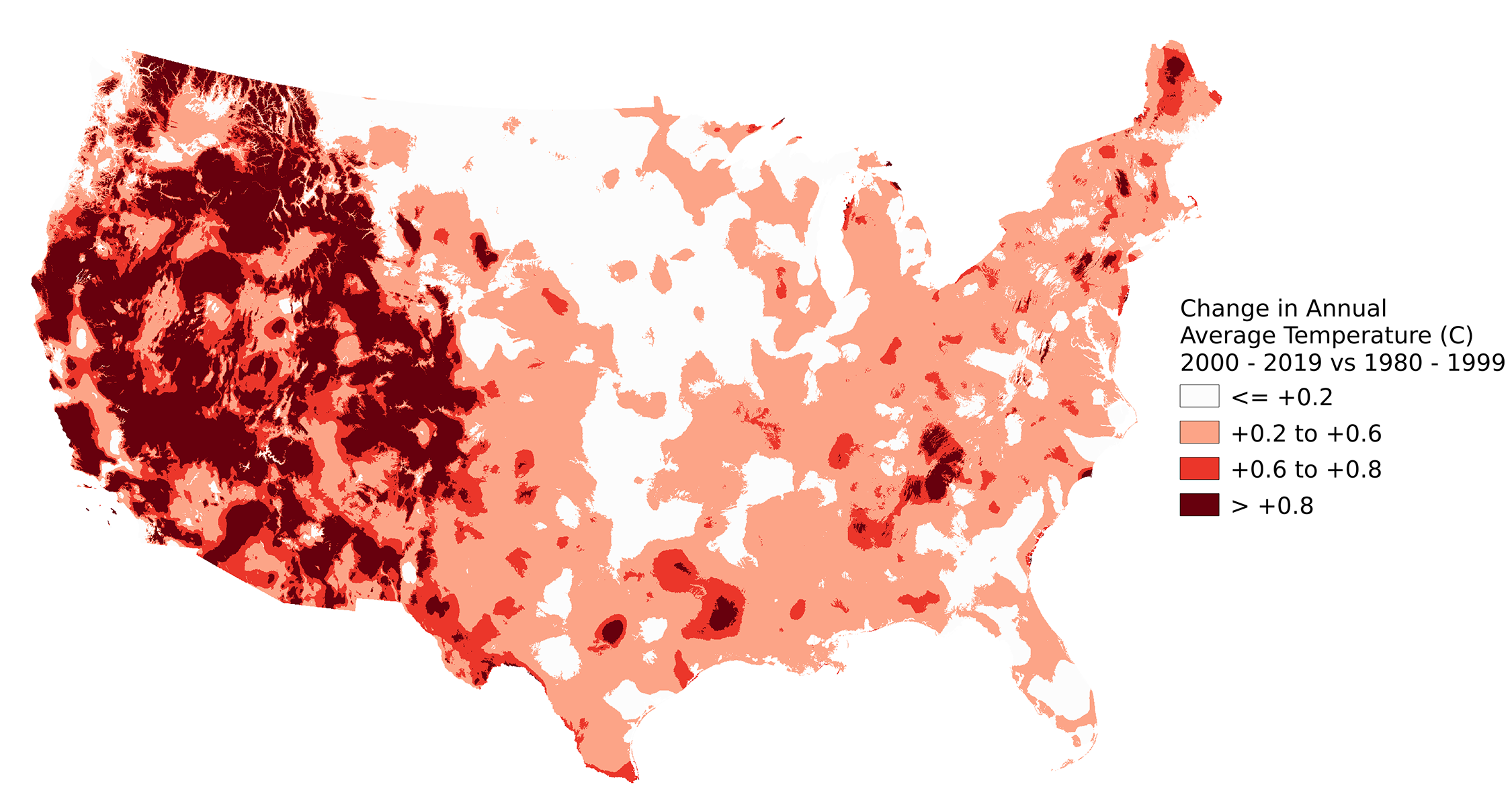

Supplement: S1 Fig — Data source = Daymet. (TIF) [file pone.0256586.s001.tif]

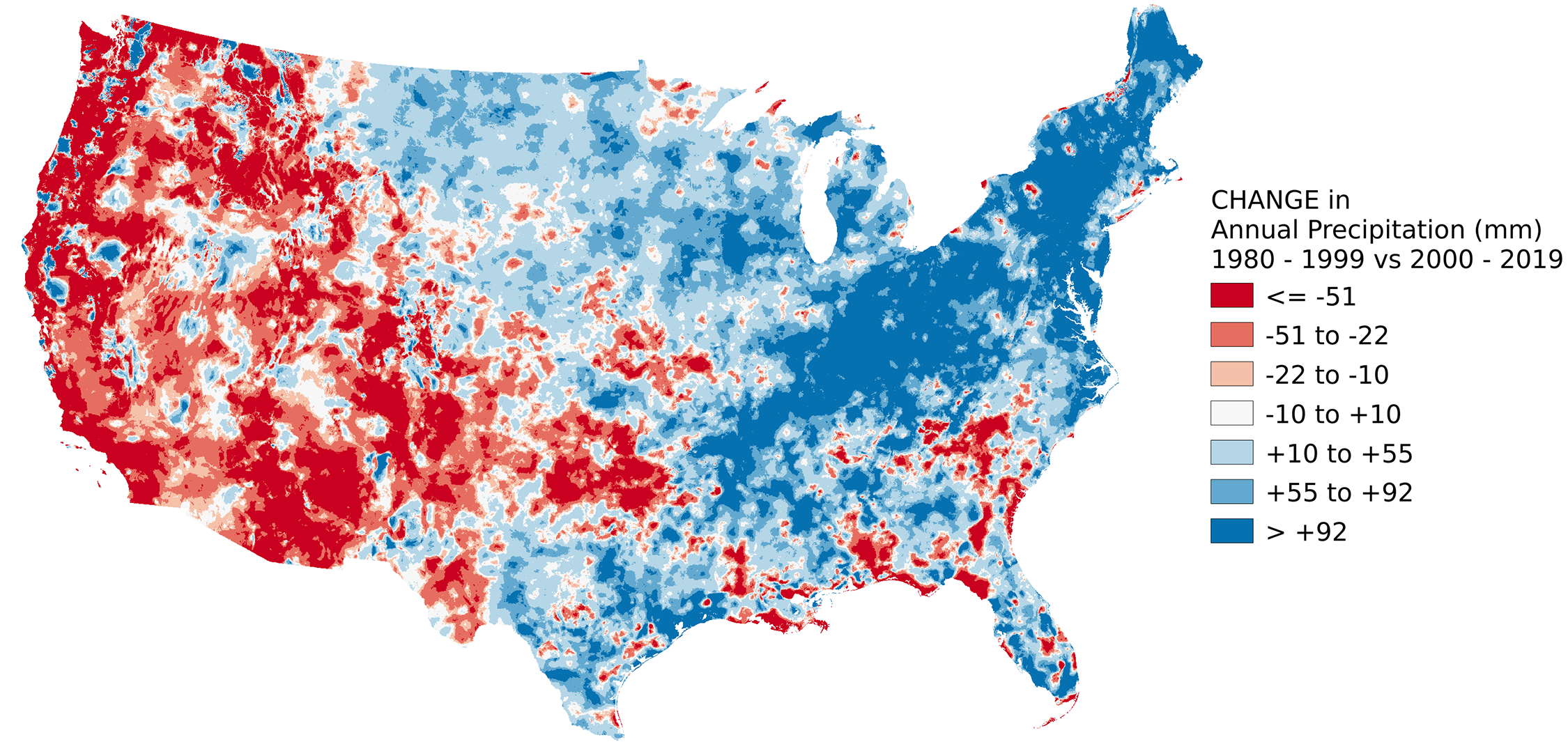

Supplement: S2 Fig — Data source = Daymet. (TIF) [file pone.0256586.s002.tif]

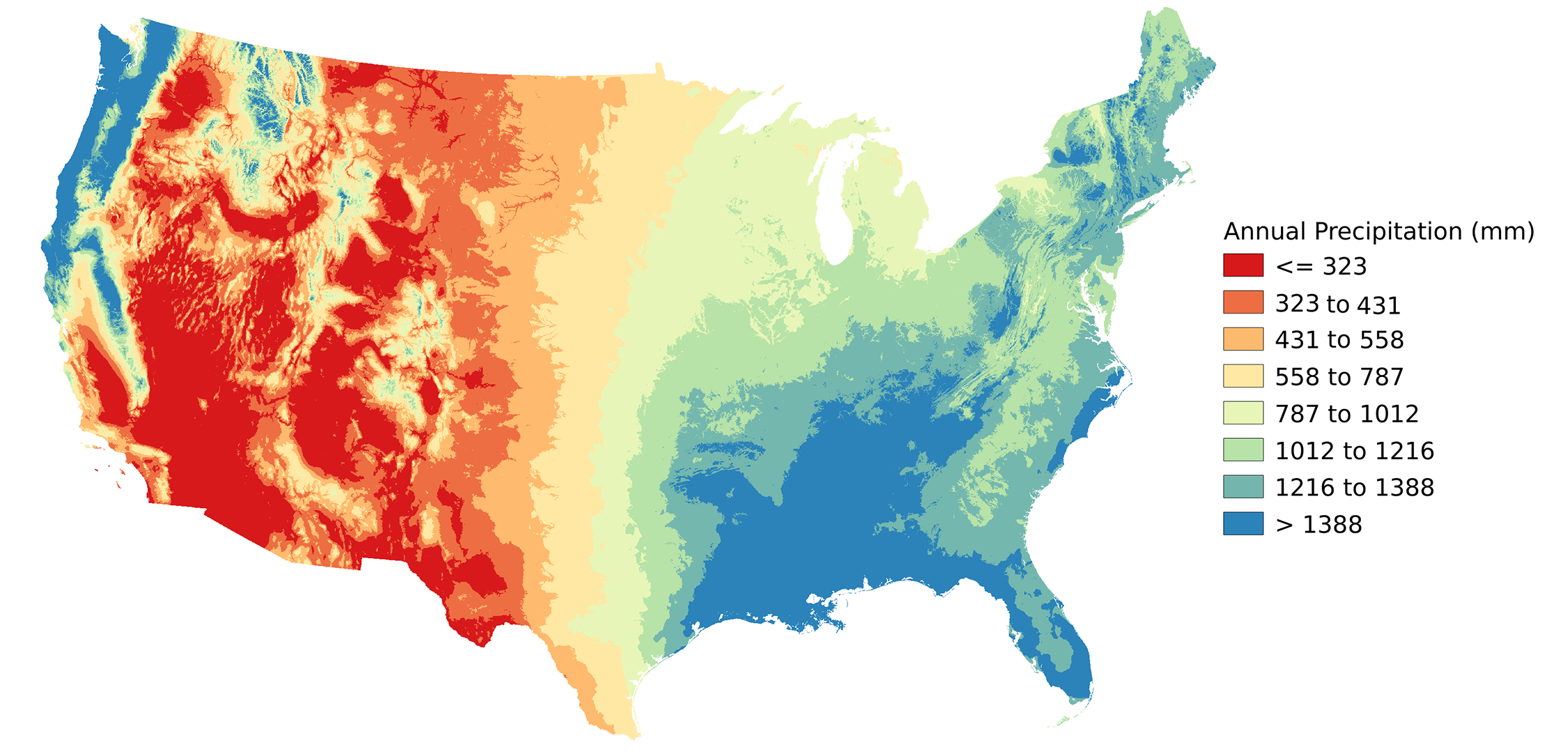

Supplement: S3 Fig — Data source = Daymet. (TIF) [file pone.0256586.s003.tif]

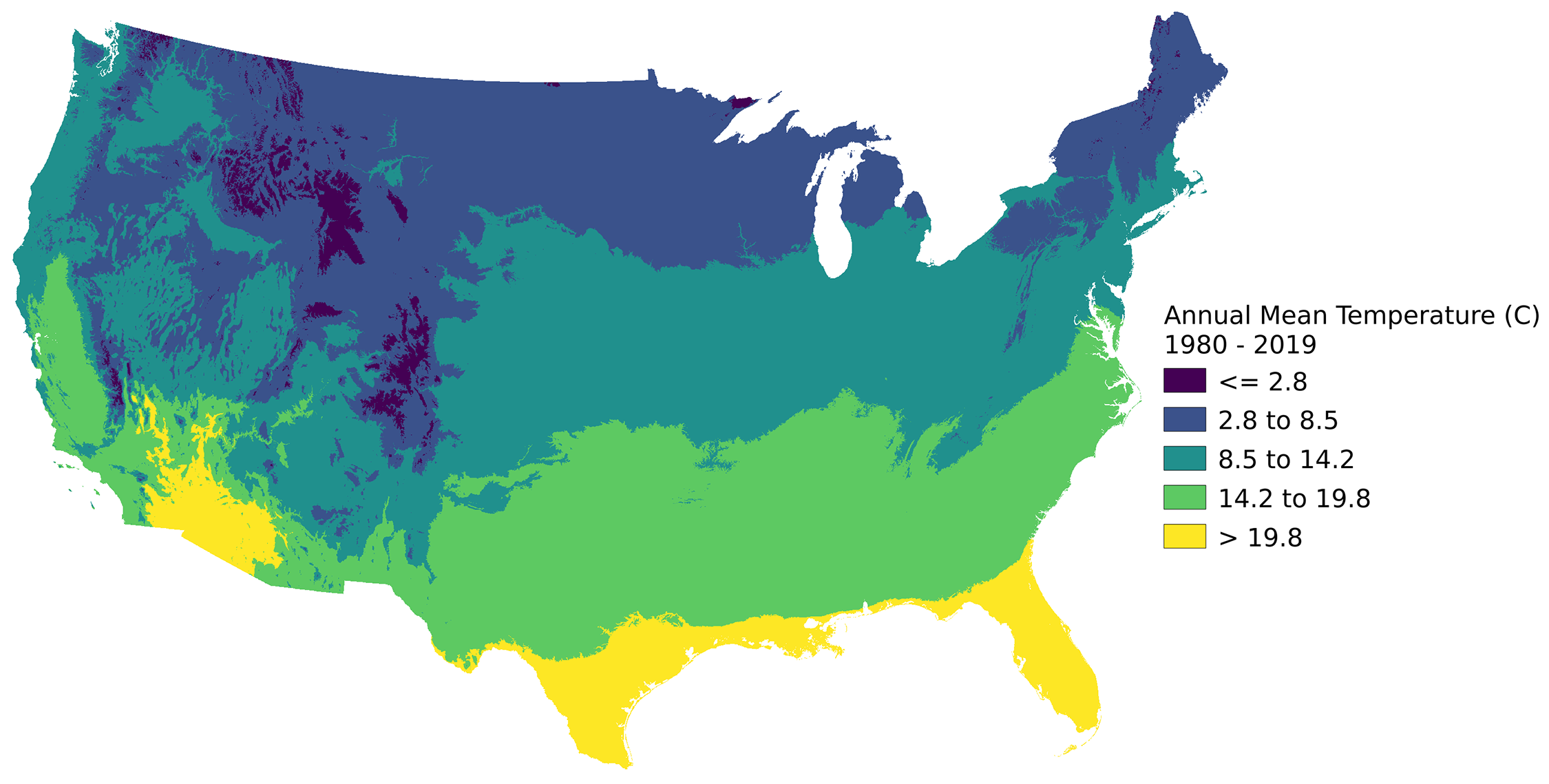

Supplement: S4 Fig — Data source = Daymet. (TIF) [file pone.0256586.s004.tif]

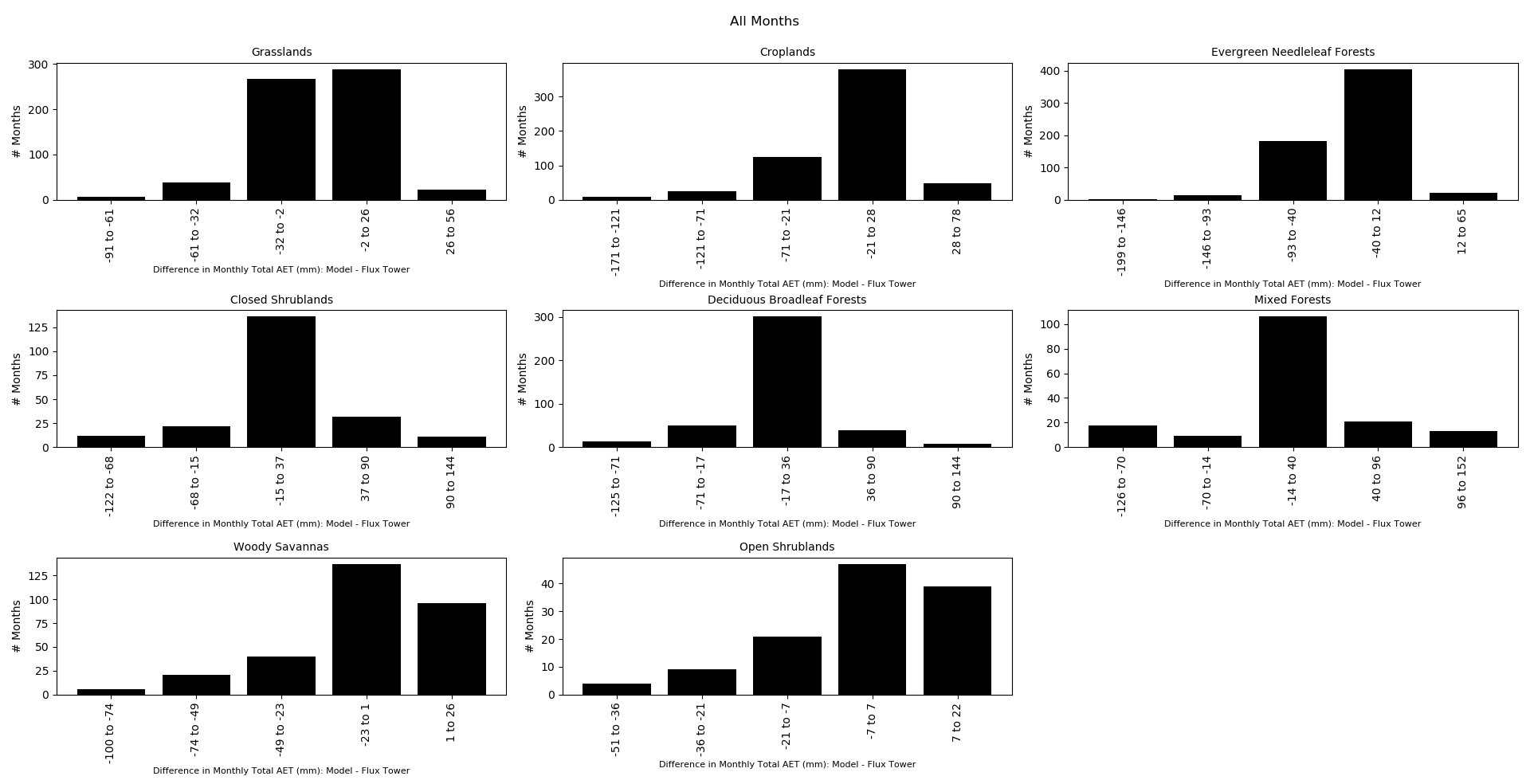

Supplement: S5 Fig — Estimates for all years are averaged for 2005–2019 before comparison to the corresponding pixel. Differences are calculated as model average–SNODAS average. (PNG) [file pone.0256586.s005.png]

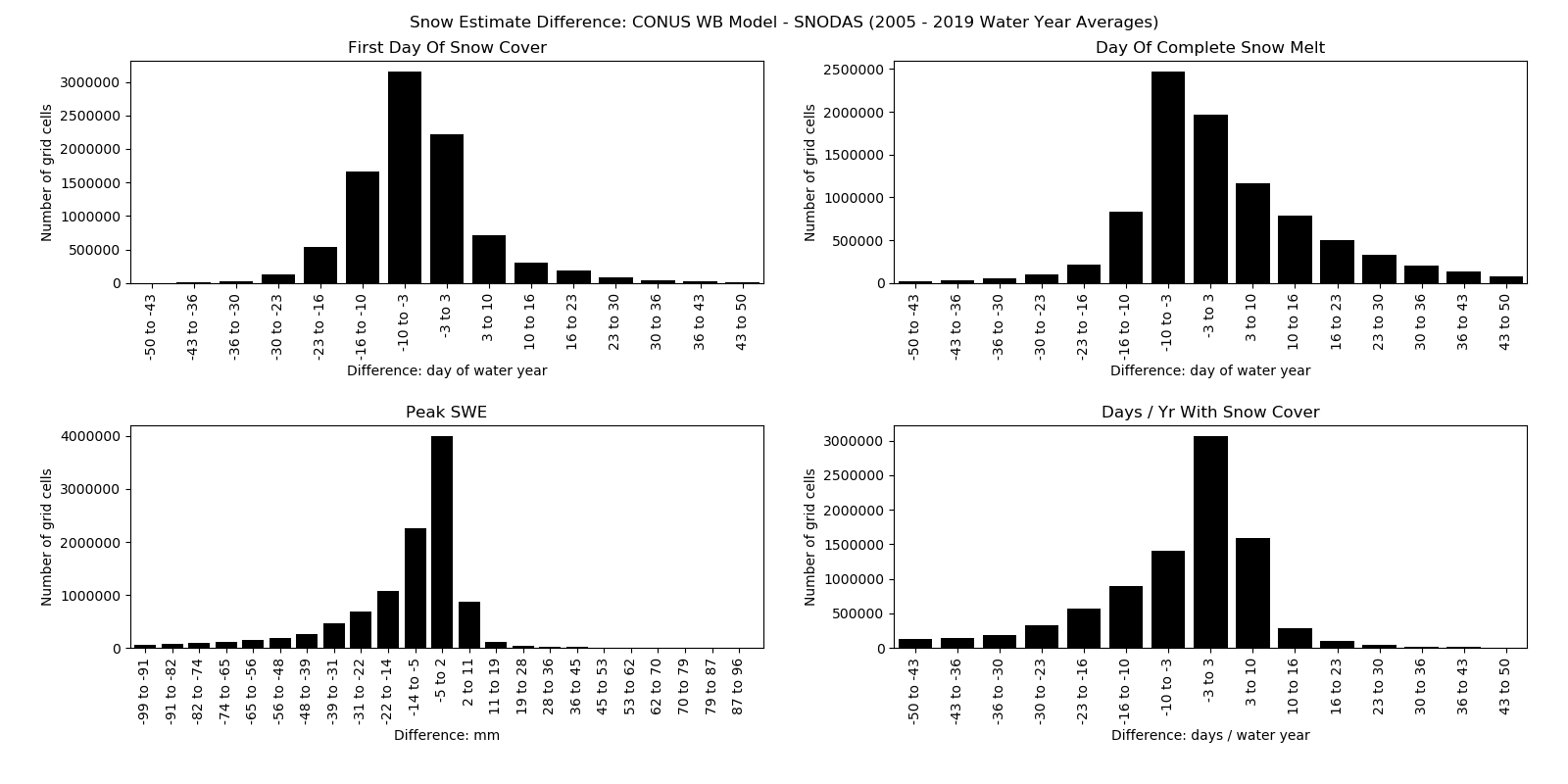

Supplement: S6 Fig — Pixels corresponding to the location of towers were extracted from the gridded dataset and classified by vegetation communities specified by fluxnet.org. Monthly differences between total AET values in the model vs at the towers were calculated over 1980–2019, or for the period of record for the flux towers, whichever was shorter. X- axis = difference in mm model AET–tower AET. Y–axis = number of months with the specified difference. (PNG) [file pone.0256586.s006.png]

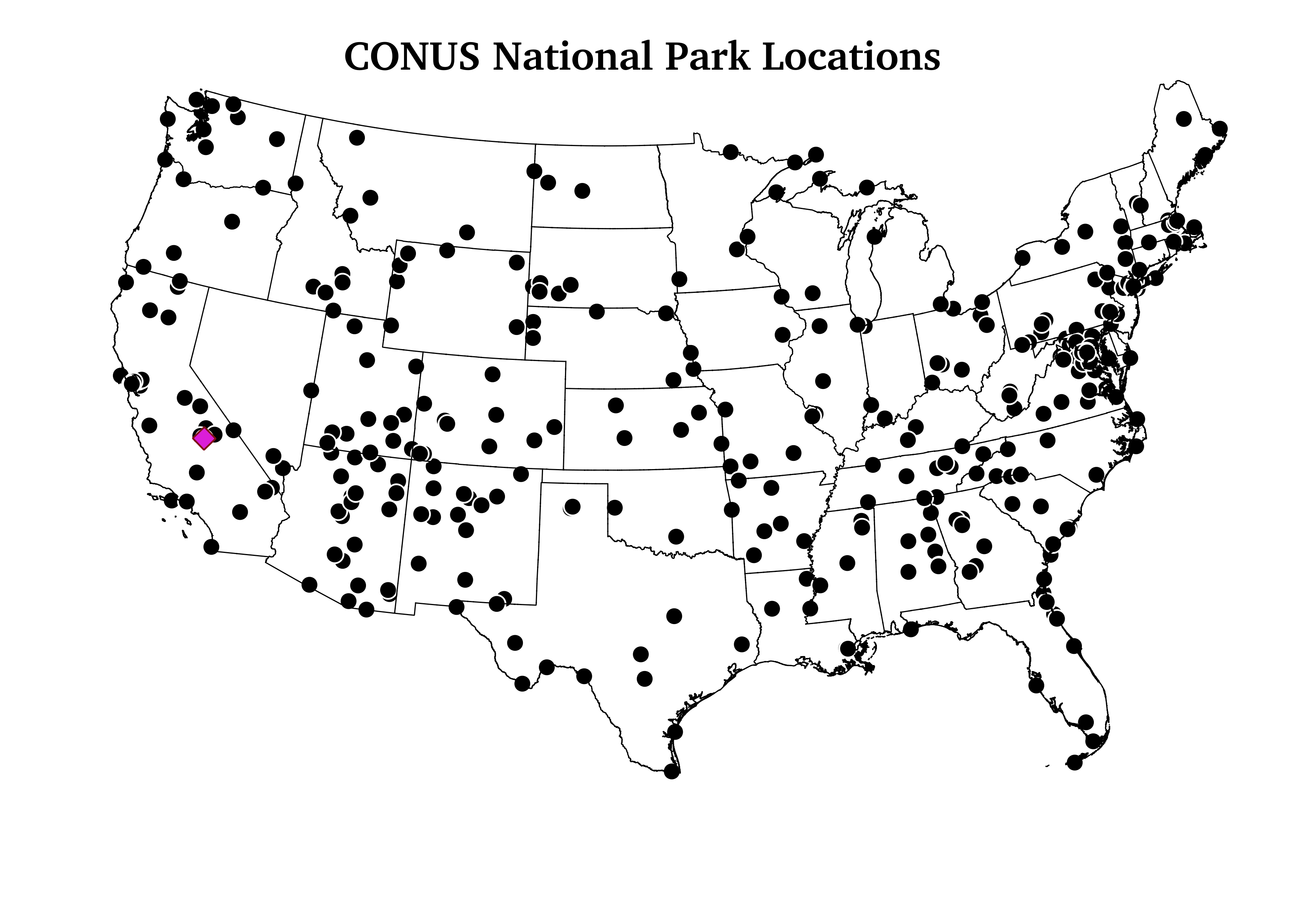

Supplement: S7 Fig — Sequoia National Park is marked with a purple diamond. (TIF) [file pone.0256586.s007.tif]
